# Functions of Shp2 in cancer

**DOI:** 10.1111/jcmm.12618

**Published:** 2015-06-19

**Authors:** Jie Zhang, Fei Zhang, Ruifang Niu

**Affiliations:** a Key Laboratory of Breast Cancer Prevention and Therapy, Ministry of Education, Key Laboratory of Cancer Prevention and Therapy, National Clinical Research Center for Cancer, Tianjin Medical University Cancer Institute and HospitalTianjin, China

**Keywords:** Shp2, cancer, invasion and metastasis, apoptosis, cell proliferation, DNA damage, drug resistance

## Abstract

Diagnostics and therapies have shown evident advances. Tumour surgery, chemotherapy and radiotherapy are the main techniques in treat cancers. Targeted therapy and drug resistance are the main focus in cancer research, but many molecular intracellular mechanisms remain unknown. Src homology region 2-containing protein tyrosine phosphatase 2 (Shp2) is associated with breast cancer, leukaemia, lung cancer, liver cancer, gastric cancer, laryngeal cancer, oral cancer and other cancer types. Signalling pathways involving Shp2 have also been discovered. Shp2 is related to many diseases. Mutations in the ptpn11 gene cause Noonan syndrome, LEOPARD syndrome and childhood leukaemia. Shp2 is also involved in several cancer-related processes, including cancer cell invasion and metastasis, apoptosis, DNA damage, cell proliferation, cell cycle and drug resistance. Based on the structure and function of Shp2, scientists have investigated specific mechanisms involved in cancer. Shp2 may be a potential therapeutic target because this phosphatase is implicated in many aspects. Furthermore, Shp2 inhibitors have been used in experiments to develop treatment strategies. However, conflicting results related to Shp2 functions have been presented in the literature, and such results should be resolved in future studies.

## Introduction

Src homology region 2 (SH2)-containing protein tyrosine phosphatase 2 (Shp2), encoded by the ptpn11gene, is a non-receptor phosphotyrosine phosphatase. Shp2 is ubiquitously expressed in various vertebrate cells. Shp2 contains one protein tyrosine phosphatase (PTP) catalytic domain and two SH2 domains. Two tandem-arranged SH2 domains are found in the N-terminal region of Shp2 and a phosphatase domain is located in the C-terminal domain of Shp2 1–3. The N-SH2 domain is a conformational switch that binds and inhibits phosphatase or binds phosphoproteins and activates enzymes; whereas the C-SH2 domain contributes binding energy and specificity; however, the C-SH2 domain does not play a direct role in activation 4. Furthermore, Shp2 contains two tyrosine residues (Y542 and Y580), which can be phosphorylated in the presence of extracellular stimulation. Bennett *et al*. 5 first identified Shp2 as a major phosphorylation site in response to platelet-derived growth factor (PDGF). Since then, many stimuli, including some cytokines and growth factors, have been found to activate Shp2. Ptpn11 is also the first identified proto-oncogene that encodes a tyrosine phosphatase 6 and it has been extensively investigated in the field of cancer. Ptpn11-related phosphatase activity is implicated in the regulation of intracellular signalling activity 7–9. Experimental and clinical data have also indicated that Shp2 promotes tumour progression in many types of cancer.

### Shp2 in different types of cancer

Shp2 is closely related to cancer; for this reason, researchers have focused on the role of Shp2 in various types of cancer, and results have shown that Shp2 may be a new target of anti-neoplastic drugs 10. Since Zhou and Agazie 11 first proposed that Shp2 is upregulated in breast cancer cells, various regulatory mechanisms of Shp2 in breast cancer have been found. For instance, Aceto *et al*. 12 screened Shp2 signature genes, which are simultaneously activated in a large subset of human primary breast tumours associated with invasive behaviour and poor prognosis; this result provided new insights into the signalling cascades influencing tumour-initiating cells and a rationale to target Shp2 in breast cancer 12. Furthermore, Shp2 interacts with growth factor receptor (GFR)-bound protein 2/Grb2 associated binding protein 1 (Grb2/Gab1) 13, participates in signal transducer and activator of transcription 1 (Stat1) regulation 14 and promotes signal transduction of breast cancer markers, such as human epidermal growth factor (EGF) receptor 2 (Her2) 15 to control tumour development. In this manner, the important position of Shp2 in breast cancer is established. Therefore, the function of this protein in gastric and lung cancers has been investigated 16,17. Shp2 may also play an important role in the progression of oral squamous cell carcinoma (OSCC) 18. Moreover, Shp2 expression is negatively correlated with patient prognosis; Shp2 further promotes tumourigenesis in laryngeal cancer; and the mitogen-activated protein kinase (MAPK) pathway is involved in Shp2-induced growth of laryngeal cancer cells 19. Another study found that Shp2 expression is also correlated with human papillomavirus infected cervical cancer 20, and it also promotes cell proliferation by inhibiting interferon (IFN)-β production 21.

Ptpn11 is a crucial oncogene that has been extensively investigated. Nevertheless, ptpn11/Shp2 exhibits a tumour-suppressing function in liver cancer 22; Yang *et al*. 23 has suggested that ptpn11 suppresses tumourigenesis in cartilage, and this finding indicated that the function of ptpn11 is tissue specific. Shp2 deficiency is oncogenic in cartilage cell population characterized by cathepsin K expression. In these cells, extracellular regulated protein kinases (Erk) normally repress the expression of the growth stimulator Indian hedgehog and the production of parathyroid hormone-related protein. Monitoring of the tumour microenvironment and other compensatory pathways should be strengthened to avoid the abuse of pathway inhibitors.

Based on the structure of Shp2, phosphorylation sites and phosphatase activity have been commonly investigated. As a phosphatase, its activity is also implicated in diverse cancers *via* Shp2 mutants. In this article, this implication is introduced. Several studies have focused on the extent of Shp2 phosphorylation activation, not its phosphatase activity. A previous report has suggested that phosphorylated Tyr 542 and Tyr 580 can interact intramolecularly with N-SH2 and C-SH2 domains, respectively; thus, this interaction, prevents basal inhibition of phosphatase activity 24. This type of relationship has established the association between phosphatase activity and Shp2 phosphorylation.

Receptor tyrosine kinase (RTK) activates a series of signal transduction pathways and affects tumour progression. Shp2 is a phosphatase; hence, RTK signalling related to this phosphatase has been commonly explored. Furthermore, previous studies demonstrated that Shp2 is a critical mediator involved in the activation of the small G protein Ras-Erk (Ras-Erk) signalling pathway 25,26.

The expression of Shp2 catalytically inactive mutant C459S inhibits Erk activation in response to insulin but not in response to 12-*O*-tetradecanoyl phorbol-13-acetate 27. Likewise, Shp2 can be phosphorylated by stimulation with growth factors or cytokines, such as EGF 28, and hepatocyte growth factor 29, to activate the Ras-Erk signalling pathway. Glycoprotein gp130 (CD130) 16 and GFR 30 interact with Shp2 to mediated signalling; as such, the mechanism by which Shp2 is phosphorylated by various exogenous stimuli has been widely studied. Furthermore, one (or both) of these tyrosines should be phosphorylated to activate Shp2. Correspondingly, Miura 29 suggested that tyrosine phosphorylation of Shp2, not phosphatase activity of Shp2 is implicated in Erk activation. The molecular mechanism of Shp2 phosphorylation activity has been investigated, particularly kinase, which stimulates Shp2 phosphorylation. EphA2 is identified as a tyrosine kinase that phosphorylates Tyr542 and Tyr580 of Shp2 to enhance and prolong Erk activation in cells stimulated by growth factors 29. Thus, whether Shp2 phosphatase activity or phosphorylation level is necessary to activate the Erk pathway remains controversial. Moreover, whether these factors cause different phenomena under various stimuli remains unknown.

Although experimental data have shown that Shp2 plays a key role in the activation of the Erk signalling, Tseng *et al*. 31 demonstrated that Shp2 influences IFN-γ resistance but does not affect hyperproliferation or Erk activation in gastric cancer by participating in PI3K-Akt signalling.

Grb2 controls fibroblast GFR 2 (FGFR2) phosphorylation by inhibiting receptor kinase and Shp2 phosphatase activity 30. Thus, Shp2 is a critical mediator of the activation of the PI3K-Akt signalling pathway 25. Epidermal growth factor induces rapid and transient interaction of Shp2 with Gab1; in turn, this interaction mediates association with EGFR and activation of PI3K 32.

Grb2 should be considered in studies in involving Shp2, because the interaction between Grb2 and Shp2 is essential to activate the downstream pathway of Erk 29,30,33. Activated Shp2 recruits Grb2; phosphorylated Tyr580 of Shp2 functions as the main binding site of Grb2, thereby activating Ras in response to growth factor 34. In addition, Grb2 controls FGFR2 phosphorylation by inhibiting receptor kinase and Shp2 phosphatase activity 30.

Activated Shp2 can downregulate tyrosine phosphorylation of Stat3, which promotes the Noonan syndrome (NS) and juvenile myelomonocytic leukaemia (JMML) 35. Although Stat3 activation is essential for cancer progression, especially breast cancer 36,37, however, Shp2 is a proto-oncogene promotes breast cancer 11; these conflicting results indicated that an unknown mechanism should be further investigated. Shou *et al*. 35 proposed that the negative regulation of Shp2-Stat3 and positive promotion of the Shp2-Ras pathways are synergistic, but the mechanism is still unclear. We deduce that Shp2 is an adaptor protein that recruits the Grb2/Sos component and activates MAPK, and the scaffolding role is dependent on tyrosine phosphorylation. Its phosphorylation enhances the phosphatase activity of Shp2, so p-Shp2 can bind to some substrates to function.

### Shp2 mutants and cancer

Shp2 mutation has been detected in several diseases, such as NS 38, childhood leukaemia, and human malignancies 39,40. Ptpn11 gene mutations are common in patients with NS and LEOPARD syndrome (LS), two developmental disorders with pleiomorphic phenotypes. These conditions are mainly caused by mutations of the ptpn11 gene that catalytically inactivates tyrosine phosphatase Shp2 in LS but activates this phosphatase in NS. Two recurrent mutations, namely, Tyr279Cys and Thr468Met 41, have also been identified in patients with LS; Thr468Met mutation is used to construct animal models of LS 42. The mutation Gln506Pro is in the PTP domain of Shp2. This region is a common site of mutation, in which Shp2 is activated to a great extent when residues directly involved in binding at the interface between the N-terminal Src homology 2 and PTP domains are altered. Such mutants prolong signal flux *via* theErk2/MAPK1 pathway; this mechanism requires docking *via* Grb2-associated binder-1 (Gab1), thereby promoting cell proliferation 43.

Shp2 mutants are related to cancer. Mutations in ptpn11 occur at low frequencies in several human cancers, particularly neuroblastoma and acute myelogenous leukaemia (AML) 44. Leukaemia-associated mutant Shp2-E76K is one of the most common and active ptpn11 mutation found in leukaemia and solid tumours. Shp2-E76K is associated with Gab1 in the lungs of transgenic mice. When activated, Shp2 mutants promote lung tumourigenesis; thus, Shp2 mutants are essential for tumour maintenance in the mouse model of non-small cell lung cancer (NSCLC) 45. PHPS1 (specific inhibitor of Shp2) efficiently inhibits Erk1/2 activation by Shp2-E76K, a leukaemia-associated Shp2 mutant, and blocks the anchorage-independent growth of various human tumour cell lines 46. Shp2-Q51E, a dominant-negative loss of function mutation, increases cell migration 47 and causes hypertrophic cardiomyopathy by dysregulating mTOR signalling.

Although several mutants, such as D61G, Y279C, N308D, T468M and E76K have been studied and compared with Shp2-N308D, Shp2-E76K possesses higher phosphatase activity 48. Moreover, the frequency of mutation in tumours is not very high. Nevertheless, this result provided the basis for studying Shp2 activity. Furthermore, gene mutation is an effective mechanism to understand gene functions. In cancer, the phosphatase activity of Shp2 is a result of mutations and may serve as a switch in different signal stimulations to reveal different pathways.

## Functions of Shp2 in cancers

### Tumour invasion and metastasis

Shp2 mediates epithelial mesenchymal transition (EMT) and is upregulated in breast cancer cells 11. Various regulatory mechanisms of Shp2 in breast cancer have also been found. Shp2 depletion prevents invasion *in vivo*, and Shp2 knockdown in established breast tumours inhibits growth and impedes metastasis 12. In triple-negative breast cancer (TNBC) cells, Shp2 affects motility *in vivo*, as well as cell migration and invasion *in vitro*, by activating several SRC-family kinases and downstream targets 49. In CXC chemokine ligand-12 -induced chemotaxis and chemoinvasion in breast cancer cells, Shp2 functions as a kind of component of the multimeric complex that mediates these processes 50. Shp2 also modulates the activity of focal adhesion kinase (Fak) by dephosphorylating pTyr397 to mediate lamellipodia persistence and cell polarity; in turn, cell migration in MDA-MB231 and MDA-MB468 basal-like and TNBC cell line is promoted 51. Shp2 overexpression is positively related to Her2 overexpression, high tumour grade and lymph node metastasis 52.

In other cancers, such as OSCC, Shp2 overexpression is associated with advanced tumour clinical stages and lymph node metastasis *ex vivo*. The knockdown of Shp2 expression *in vitro* inhibits OSCC cell viability and invasion 18. Furthermore, Shp2 promotes invasion and metastasis of oral cancer cells; this result indicated that the Shp2-ERK1/2-Snail/Twist1 pathway is possibly implicated in oral cancer invasion and metastasis 53. Clinical data have also suggested that Shp2 expression in NSCLC exhibits high specificity and sensitivity, and this expression is closely related to lymph node metastasis. Shp2 expression may promote invasion and metastasis of NSCLC *via* angiogenesis and *via* the lymphatic system 54,55. Transforming growth factor-β1-induced EMT in lung epithelial A549 cells is partially blocked when Shp2 is decreased by transfected siRNA; tyrosine phosphatase Shp2 is involved in EMT, and Hook1 represses EMT by regulating Shp2 activation. The Shp2-Hook1 (hook microtubule-tethering protein 1) complex may also play important roles in tumour metastases by regulating EMT 56. The mRNA levels of Shp2 are significantly higher in gastric cancer tissues than those in normal gastric mucosa. In addition, Shp2 expression is significantly correlated with tumour differentiation, clinical classification and lymph node metastasis 57. The migration of anaplastic large cell lymphoma cells is reduced by Shp2 shRNA. These findings showed that Shp2 is directly involved in nucleophosmin/anaplastic lymphoma kinase lymphomagenesis, highlighting its critical role in lymphoma cell proliferation and migration 58. Moreover, the interaction with Y580-Shp2 localizes Fyn to receptor sites required for α6β4-dependent carcinoma invasion 59. The knockdown of Shp2 significantly increases podosome rosette formation in Src-transformed fibroblasts by selectively suppressing the tyrosine phosphorylation of Src substrate Tks5, a scaffolding protein necessary to form podosome 60; this finding may elucidate the mechanism by which tumour metastasis is promoted.

### Apoptosis in cancer

In cancer research, the apoptotic role of Shp2 was first discovered in multiple myeloma cells. Chauhan *et al*. 61,62 demonstrated that activated Shp2 inhibits the activation of a related adhesion focal tyrosine kinase, also known as Pyk2, and Shp2 is involved in the process by which interleukin-6 (IL-6) blocks apoptosis induced by dexamethasone. Leukaemia, a Shp2-associated disease, has been intensively investigated. The suppression of Shp2 expression induces apoptosis and inhibits leukaemic cell clonogenic growth 63. In addition, the knockdown of Shp2 expression *in vitro* induces OSCC cell apoptosis by regulating the expression of apoptosis-related proteins 18. The activation of Shp2 PTP is synergized with IFN consensus sequence binding protein haploinsufficiency to facilitate cytokine-induced myeloproliferation, apoptosis resistance and rapid progression to AML in a murine bone marrow transplantation model 64.

Yang *et al*. 65,66 showed that tyrosine phosphatase Shp2 prevents apoptosis in tumour stem cells by activating Erk. The expression of gain of function (GOF) mutation Shp2-E76K, the most common and active ptpn11mutation found in leukaemia and solid tumours, suppresses the apoptosis pathway 67. Shp2-D61Y or Shp2-E76K-expressing hematopoietic cells also reduce apoptosis, as indicated by Annexin-V staining results, and they produces increased progenitor colonies after 48 hrs in minimal media compared with cells transduced with an empty vector or wildtype of Shp2 68. These results proved that the phosphatase activity of Shp2 plays a key role in controlling apoptosis.

Shp2 is associated with apoptosis suppression. However, liver cancer cells showed anoikis when treated with arecoline; furthermore, IL-6 expression and Stat3 phosphorylation provide protection against anoikis; caspase-3 activity is increased and Shp2 is inhibited by arecoline 69. In another study, the knockdown of Shp2 inhibits sorafenib-induced Tyr(705) p-Stat3 dephosphorylation and increases tumour cell apoptosis in cholangiocarcinoma cells 70. These results were consistent with that of Feng *et al*. 22, who demonstrated that ptpn11/Shp2acts as a tumour suppressor in hepatocellular carcinogenesis. These conflicting results are observed in a U2OS osteosarcoma cell line, in which the knockdown of Shp2 expression with small interfering RNA in apoptotic cells increases cell viability and rescues cells from retinoblastoma/transcription factor E2F-associated apoptotic response to inhibition of cleavage of both caspase-8 and caspase-3 71. Tumour necrosis factor-induced EC apoptosis is possibly reduced significantly in Shp2-knockout EC by regulating apoptosis signal-regulating kinase 1 phosphorylation and stability in response to cytokines 72.

In addition, Shp2 plays an essential role in controlling the survival and maintenance of hematopoietic stem cells by decreasing apoptosis *in vivo* 73,74. Shp2-knockout mice exhibit a remarkable reduction in surfactant proteins with increased alveolar epithelial apoptosis 75. Furthermore, Cat.G induces Shp2 activation that leads to Fak tyrosine dephosphorylation and promotes cardiomyocyte anoikis 76. The inactivation of Shp2 sensitizes cells to epigallocatechin gallate (EGCG)-mediated death, and mouse embryonic fibroblasts without functional Shp2 undergo massive apoptosis after these factors are treated with EGCG; thus, Shp2 serves as a negative regulator of EGCG-induced apoptosis 77. Shp2 may contribute to Erk5 activation by participating in Src kinase activation and by docking to PDGF receptor beta, such that PDGF-BB fails to suppress caspase-3 activation and inhibit apoptotic nuclear morphological changes 78. Shp2 is needed to prevent pulmonary arterial hypertension-pulmonary artery smooth muscle cells apoptosis 79. These results may help explain Shp2-regulated apoptosis.

### Tumour cell proliferation and cell cycle

Shp2 is demonstrated as a tumour-promoting gene by regulating invasion and apoptosis; Shp2 is also a factor that promotes cell proliferation 80–82. In cancer, Shp2 regulates multivariate signalling regulation to control proliferation in glioma cells 83. Tyrosine phosphatase Shp2 also promotes the proliferation of breast carcinoma 84. These phenomena provide the basis for investigating the role of Shp2 in the cell cycle.

Yang *et al*. 68 suggested that GOF Shp2 mutants promote hematopoietic progenitor cell cycle progression and survival. In another report, agents targeting cell cycle or promoting apoptosis were found to have therapeutic potential in JMML. Microinjection of antibodies can block the interaction of the SH2 domains of the PI3K p85α subunit with tyrosine phosphorylated intracellular targets that inhibit DNA synthesis; thus, when antibodies to Shp2 are injected during the first 15 min. of the G1 phase, DNA synthesis is inhibited 85. Shp2 promotes the growth of glioblastoma cells by suppressing cellular senescence 86. Shp2 and Stat5 function as proximal effectors of the Kit oncogene, and cell survival is driven by the Shp2/Erk pathway; conversely, G1/S transition during the cell cycle is accelerated by the Kit/Stat5 and Kit/PI3K/Akt pathways 87. Shp2 is also involved in radioresistance by controlling cell cycle distribution in nasopharyngeal carcinoma cell lines 88. In HeLa cell line, Shp2 depletion arrests checkpoint-mediated cell; these results indicated the importance of Shp2 in checkpoint control and revealed a novel link between Shp2 and cell cycle checkpoints 89. These findings may also explain the function of Shp2 in cell proliferation and provide a new direction for novel drug research; however, additional evidence should be obtained to support these data.

### DNA damage and replication in cancer

The DNA replication function of Shp2 is still a new research direction. In a previous study, ptpn11 (Shp2) is involved in the p53 pathway, including anti-apoptotic pathways, structural loss, and DNA replication 90. Further detailed studies have revealed that Shp2 is necessary to maintain checkpoints after DNA damage is induced by cisplatin or ionizing radiation in HeLa cells; Shp2 is also activated after cells are exposed to replicative stress and DNA damage, and Shp2 depletion impairs checkpoint kinase 1 activation and checkpoint-mediated DNA repair 89. In another study, Shp2 is related to DNA damage 45G (GADD45G), a stress sensor with multiple implications in various biological processes; this stress sensor is downregulated in a broad spectrum of cancers. The ectopic expression of GADD45G induces senescence in hepatocellular carcinoma (HCC) cells and suppresses tumour growth *in vivo*. The knockdown of Shp2 efficiently counteracts GADD45G-induced senescence. In clinical HCC specimens, GADD45G expression is inversely correlated with phosphorylated Stat3 expression in tumour cells and disease progression 91; this result was consistent with the relationship of Shp2 with Stat3 36,37.

### Drug resistance in cancer

Shp2 is involved in the treatment of the EGFR signalling pathway of H1975 cells, in which II-B08, a specific Shp2 inhibitor, is used to reduce Erk1/2 activation; therefore targeting Shp2 may represent an effective strategy for the treatment of EGFR inhibitor resistant NSCLCs 92. Gastric cancer cell line AGS does not respond to IFN-γ, and PI3K/AKT mediates IFN-γ resistance. Tseng *et al*. 31 reported that IFN-γ resistance is regulated by Pten/Akt/GSK-3β/Shp2 signalling in hyperproliferating gastric cancer cells.

Traditional radiotherapy and chemotherapy are modestly effective cancer treatments; nevertheless, recent advances in targeted therapies have provided a noticeable benefit to patients. However, patients eventually develop resistance to drugs. Combination therapy directed to a complementing target may significantly improve treatment results. Although the role of Shp2 in drug resistance has been partially revealed, Shp2 is involved in key signalling pathways. Therefore, the inhibitor of Shp2 may be used in combination therapy drugs.

## Concluding remarks

**Figure 1:**
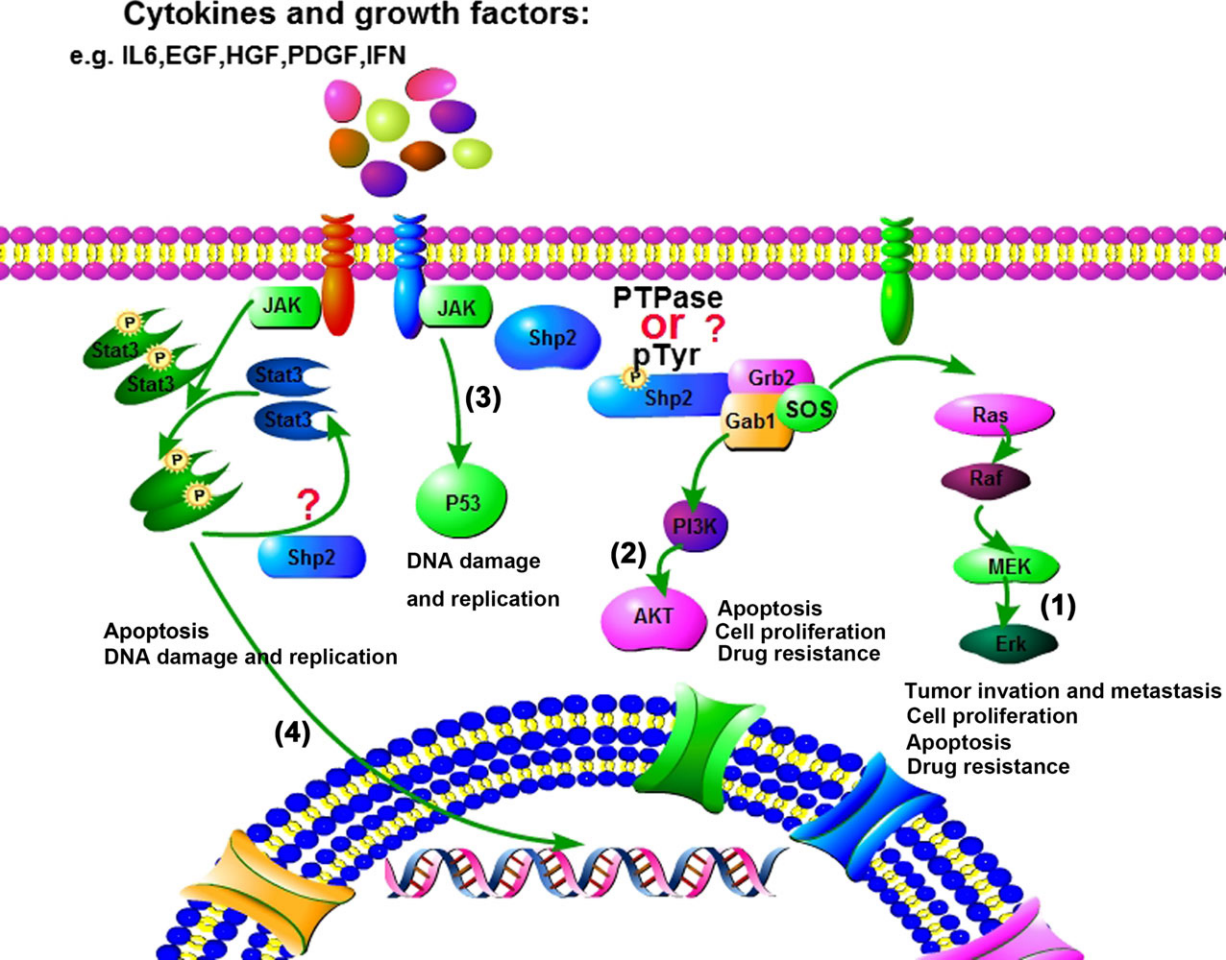
Schematic diagram of the signalling and functions of Shp2 (1). In the presence of extracellular stimulation, including some cytokines and growth factors [*e.g*. interleukin (IL)6, epidermal growth factor (EGF), hepatocyte growth factor (HGF), platelet-derived growth factor (PDGF), IFN), they binds to relevant receptor to activate the down-stream, as a result, Shp2 can be phosphorylated by the receptor tyrosine kinase (RTK), p-Shp2 binds to the Grb2/SOS to activate the Ras/Erk signalling, so that to enhance tumour invasion and metastasis. PTP activation and pTyr of Shp2 are the focus of research work, with a lot of conflicted results, it is not sure how Shp2 functions in different microenvironments, although signalling molecular inhibitors can treat and work partly, but it is necessary to ensure the safety and control the compensatory. (2) Gab1 can bind to Shp2 and activate the PI3K/Akt signalling to regulate tumour cell proliferation, tumour apoptosis and drug resistance. And still, the phosphatase activity of Shp2 plays a critical role in controlling these processes. (3) Shp2 participates in p53 signalling to regulate DNA damage and replication in cancer. (4) Stat3 can be phosphorylated to form dimer, Shp2 can dephosphorylate Stat3, but p-Stat3 is important for tumour progress, so the relationship and mechanism between p-Stat3 and Shp2 should be further investigated, and gives a more reasonable explaining for future research.

Cancer poses health risks to humans. In cancer, Shp2 plays different roles in various tumours and different microenvironments. Although great progress has been observed in studies focusing on Shp2-related mechanisms, specific processes involved in such mechanisms should be further investigated. Taken together, Figure1 is the summary of signalling pathways and functions of Shp2.

Based on the role of Shp2 in tumours, various Shp2 inhibitors have been discovered to target Shp2 for cancer treatments. Inhibitors of tyrosine phosphatase Shp2 have been widely studied because of its broad role in cancer. For instance, cryptotanshinone can be potentially used directly or developed to treat ptpn11-associated malignancies, mouse myeloid progenitors and leukaemic cells caused by E76K mutation are sensitive to this inhibitor 93. II-B08 can inhibit Shp2 and strongly bind to the receptor 94. Shp2 inhibitor II-B08 enhances the effects of dasatinib on human and mouse mastocytoma cells 95. Furthermore, computer-aided drug designs are used to discover Shp2 inhibitors 96. Shp2 inhibitors play only a partial role, but these inhibitors have shown promising results for developing drugs to treat cancers.

Although the detailed mechanism of Shp2 in cancer progression needs further investigation and the activity of Shp2 in tumours has been analysed to provide a theoretical basis for cancer treatment, better research ideas and more definitive results may help develop successful therapeutic strategies for this deadly disease. Targeted studies have revealed that a combination of inhibitors may be required to effectively block a given function in cancer research. Studies that broaden our understanding of the functions of Shp2 could lead to a re-evaluation of the role in determining clinical outcome. However, future studies of the clinical importance should be carefully designed to explain conflicting viewpoints. Drugs should be used with caution as a result of the different functions of Shp2 in various signalling pathways and cancer types. Ultimately, future studies should focus on confirming the effects of Shp2 on tumours in different tumour micro-environments, as well as the signalling pathway, including the substrate of Shp2 phosphatase activity. The overall effects of microenvironments should be studied by combining several factors.

## References

[b1] Feng GS, Shen R, Heng HH (1994). Receptor-binding, tyrosine phosphorylation and chromosome localization of the mouse SH2-containing phosphotyrosine phosphatase Syp. Oncogene.

[b2] Feng GS, Hui CC, Pawson T (1993). SH2-containing phosphotyrosine phosphatase as a target of protein-tyrosine kinases. Science.

[b3] Feng GS, Pawson T (1994). Phosphotyrosine phosphatases with SH2 domains: regulators of signal transduction. Trends Genet.

[b4] Hof P, Pluskey S, Dhe-Paganon S (1998). Crystal structure of the tyrosine phosphatase SHP-2. Cell.

[b5] Bennett AM, Tang TL, Sugimoto S (1994). Protein-tyrosine-phosphatase SHPTP2 couples platelet-derived growth factor receptor beta to Ras. Proc Natl Acad Sci USA.

[b6] Chan RJ, Feng GS (2007). PTPN11 is the first identified proto-oncogene that encodes a tyrosine phosphatase. Blood.

[b7] Holgado-Madruga M, Emlet DR, Moscatello DK (1996). A Grb2-associated docking protein in EGF- and insulin-receptor signalling. Nature.

[b8] Kouhara H, Hadari YR, Spivak-Kroizman T (1997). A lipid-anchored Grb2-binding protein that links FGF-receptor activation to the Ras/MAPK signaling pathway. Cell.

[b9] Xu D, Wang S, Yu WM (2010). A germline gain-of-function mutation in Ptpn11 (Shp-2) phosphatase induces myeloproliferative disease by aberrant activation of hematopoietic stem cells. Blood.

[b10] Mohi MG, Neel BG (2007). The role of Shp2 (PTPN11) in cancer. Curr Opin Genet Dev.

[b11] Zhou XD, Agazie YM (2008). Inhibition of SHP2 leads to mesenchymal to epithelial transition in breast cancer cells. Cell Death Differ.

[b12] Aceto N, Sausgruber N, Brinkhaus H (2012). Tyrosine phosphatase SHP2 promotes breast cancer progression and maintains tumor-initiating cells *via* activation of key transcription factors and a positive feedback signaling loop. Nat Med.

[b13] Zhang X, Lavoie G, Fort L (2013). Gab2 phosphorylation by RSK inhibits Shp2 recruitment and cell motility. Mol Cell Biol.

[b14] Mariotto S, Ciampa AR, de Prati AC (2008). Aqueous extract of Arbutus unedo inhibits STAT1 activation in human breast cancer cell line MDA-MB-231 and human fibroblasts through SHP2 activation. Med Chem.

[b15] Zhou X, Agazie YM (2009). Molecular mechanism for SHP2 in promoting HER2-induced signaling and transformation. J Biol Chem.

[b16] Lee IO, Kim JH, Choi YJ (2010). Helicobacter pylori CagA phosphorylation status determines the gp130-activated SHP2/ERK and JAK/STAT signal transduction pathways in gastric epithelial cells. J Biol Chem.

[b17] Lazzara MJ, Lane K, Chan R (2010). Impaired SHP2-mediated extracellular signal-regulated kinase activation contributes to gefitinib sensitivity of lung cancer cells with epidermal growth factor receptor-activating mutations. Cancer Res.

[b18] Xie H, Huang S, Li W (2014). Upregulation of Src homology phosphotyrosyl phosphatase 2 (Shp2) expression in oral cancer and knockdown of Shp2 expression inhibit tumor cell viability and invasion *in vitro*. Oral Surg Oral Med Oral Pathol Oral Radiol.

[b19] Gu J, Han T, Ma RH (2014). SHP2 promotes laryngeal cancer growth through the Ras/Raf/Mek/Erk pathway and serves as a prognostic indicator for laryngeal cancer. Int J Oncol.

[b20] Meng F, Zhao X, Zhang S (2012). Expression and significance of SHP-2 in human papillomavirus infected cervical cancer. J Huazhong Univ Sci Technolog Med Sci.

[b21] Meng F, Zhao X, Zhang S (2013). SHP-2 phosphatase promotes cervical cancer cell proliferation through inhibiting interferon-beta production. J Obstet Gynaecol Res.

[b22] Bard-Chapeau EA, Li S, Ding J (2011). Ptpn11/Shp2 acts as a tumor suppressor in hepatocellular carcinogenesis. Cancer Cell.

[b23] Yang W, Wang J, Moore DC (2013). Ptpn11 deletion in a novel progenitor causes metachondromatosis by inducing hedgehog signalling. Nature.

[b24] Lu W, Gong D, Bar-Sagi D (2001). Site-specific incorporation of a phosphotyrosine mimetic reveals a role for tyrosine phosphorylation of SHP-2 in cell signaling. Mol Cell.

[b25] Agazie YM, Movilla N, Ischenko I (2003). The phosphotyrosine phosphatase SHP2 is a critical mediator of transformation induced by the oncogenic fibroblast growth factor receptor 3. Oncogene.

[b26] Maroun CR, Naujokas MA, Holgado-Madruga M (2000). The tyrosine phosphatase SHP-2 is required for sustained activation of extracellular signal-regulated kinase and epithelial morphogenesis downstream from the met receptor tyrosine kinase. Mol Cell Biol.

[b27] Noguchi T, Matozaki T, Horita K (1994). Role of SH-PTP2, a protein-tyrosine phosphatase with Src homology 2 domains, in insulin-stimulated Ras activation. Mol Cell Biol.

[b28] Paruchuri V, Prasad A, McHugh K (2008). S100A7-downregulation inhibits epidermal growth factor-induced signaling in breast cancer cells and blocks osteoclast formation. PLoS ONE.

[b29] Miura K, Wakayama Y, Tanino M (2013). Involvement of EphA2-mediated tyrosine phosphorylation of Shp2 in Shp2-regulated activation of extracellular signal-regulated kinase. Oncogene.

[b30] Ahmed Z, Lin CC, Suen KM (2013). Grb2 controls phosphorylation of FGFR2 by inhibiting receptor kinase and Shp2 phosphatase activity. J Cell Biol.

[b31] Tseng PC, Huang WC, Chen CL (2012). Regulation of SHP2 by PTEN/AKT/GSK-3beta signaling facilitates IFN-gamma resistance in hyperproliferating gastric cancer. Immunobiology.

[b32] Diaz ME, Gonzalez L, Miquet JG (2012). Growth hormone modulation of EGF-induced PI3K-Akt pathway in mice liver. Cell Signal.

[b33] Sha F, Gencer EB, Georgeon S (2013). Dissection of the BCR-ABL signaling network using highly specific monobody inhibitors to the SHP2 SH2 domains. Proc Natl Acad Sci USA.

[b34] Vogel W, Ullrich A (1996). Multiple *in vivo* phosphorylated tyrosine phosphatase SHP-2 engages binding to Grb2 *via* tyrosine 584. Cell Growth Differ.

[b35] Zhang W, Chan RJ, Chen H (2009). Negative regulation of Stat3 by activating PTPN11 mutants contributes to the pathogenesis of Noonan syndrome and juvenile myelomonocytic leukemia. J Biol Chem.

[b36] Gough DJ, Corlett A, Schlessinger K (2009). Mitochondrial STAT3 supports Ras-dependent oncogenic transformation. Science.

[b37] Wang YQ, Zhang F, Tian R (2012). Tyrosine 23 phosphorylation of annexin A2 promotes proliferation, invasion, and Stat3 phosphorylation in the nucleus of human breast cancer SK-BR-3 cells. Cancer Biol Med.

[b38] Tartaglia M, Mehler EL, Goldberg R (2001). Mutations in PTPN11, encoding the protein tyrosine phosphatase SHP-2, cause Noonan syndrome. Nat Genet.

[b39] Tartaglia M, Gelb BD (2005). Germ-line and somatic PTPN11 mutations in human disease. Eur J Med Genet.

[b40] Bennett AM, Hausdorff SF, O’Reilly AM (1996). Multiple requirements for SHPTP2 in epidermal growth factor-mediated cell cycle progression. Mol Cell Biol.

[b41] Conti E, Dottorini T, Sarkozy A (2003). A novel PTPN11 mutation in LEOPARD syndrome. Hum Mutat.

[b42] Tajan M, Batut A, Cadoudal T (2014). LEOPARD syndrome-associated SHP2 mutation confers leanness and protection from diet-induced obesity. Proc Natl Acad Sci USA.

[b43] Fragale A, Tartaglia M, Wu J (2004). Noonan syndrome-associated SHP2/PTPN11 mutants cause EGF-dependent prolonged GAB1 binding and sustained ERK2/MAPK1 activation. Hum Mutat.

[b44] Bentires-Alj M, Paez JG, David FS (2004). Activating mutations of the noonan syndrome-associated SHP2/PTPN11 gene in human solid tumors and adult acute myelogenous leukemia. Cancer Res.

[b45] Schneeberger VE, Luetteke N, Ren Y (2014). SHP2E76K mutant promotes lung tumorigenesis in transgenic mice. Carcinogenesis.

[b46] Hellmuth K, Grosskopf S, Lum CT (2008). Specific inhibitors of the protein tyrosine phosphatase Shp2 identified by high-throughput docking. Proc Natl Acad Sci USA.

[b47] Edwards MA, Crombie K, Schramm C, Krenz M (2014). The Q510E mutation in Shp2 perturbs heart valve development by increasing cell migration. J Appl Physiol.

[b48] Oishi K, Gaengel K, Krishnamoorthy S (2006). Transgenic Drosophila models of Noonan syndrome causing PTPN11 gain-of-function mutations. Hum Mol Genet.

[b49] Sausgruber N, Coissieux MM, Britschgi A (2014). Tyrosine phosphatase SHP2 increases cell motility in triple-negative breast cancer through the activation of SRC-family kinases. Oncogene.

[b50] Fernandis AZ, Prasad A, Band H (2004). Regulation of CXCR4-mediated chemotaxis and chemoinvasion of breast cancer cells. Oncogene.

[b51] Hartman ZR, Schaller MD, Agazie YM (2013). The tyrosine phosphatase SHP2 regulates focal adhesion kinase to promote EGF-induced lamellipodia persistence and cell migration. Mol Cancer Res.

[b52] Zhou X, Coad J, Ducatman B (2008). SHP2 is up-regulated in breast cancer cells and in infiltrating ductal carcinoma of the breast, implying its involvement in breast oncogenesis. Histopathology.

[b53] Wang HC, Chiang WF, Huang HH (2014). Src-homology 2 domain-containing tyrosine phosphatase 2 promotes oral cancer invasion and metastasis. BMC Cancer.

[b54] Tang C, Luo D, Yang H (2013). Expression of SHP2 and related markers in non-small cell lung cancer: a tissue microarray study of 80 cases. Appl Immunohistochem Mol Morphol.

[b55] Tang C, Zhou X, Yang H (2010). Expression and its clinical significance of SHP2 in non-small cell lung cancer. Zhongguo Fei Ai Za Zhi.

[b56] Li S, Wang L, Zhao Q (2014). SHP2 Positively Regulates TGFbeta1-induced Epithelial-mesenchymal Transition Modulated by Its Novel Interacting Protein Hook1. J Biol Chem.

[b57] Dong S, Li FQ, Zhang Q (2012). Expression and clinical significance of SHP2 in gastric cancer. J Int Med Res.

[b58] Voena C, Conte C, Ambrogio C (2007). The tyrosine phosphatase Shp2 interacts with NPM-ALK and regulates anaplastic lymphoma cell growth and migration. Cancer Res.

[b59] Yang X, Dutta U, Shaw LM (2010). SHP2 mediates the localized activation of Fyn downstream of the alpha6beta4 integrin to promote carcinoma invasion. Mol Cell Biol.

[b60] Pan YR, Cho KH, Lee HH (2013). Protein tyrosine phosphatase SHP2 suppresses podosome rosette formation in Src-transformed fibroblasts. J Cell Sci.

[b61] Chauhan D, Pandey P, Hideshima T (2000). SHP2 mediates the protective effect of interleukin-6 against dexamethasone-induced apoptosis in multiple myeloma cells. J Biol Chem.

[b62] Anderson KC (2001). Multiple Myeloma. Advances in disease biology: therapeutic implications. Semin Hematol.

[b63] Xu R, Yu Y, Zheng S (2005). Overexpression of Shp2 tyrosine phosphatase is implicated in leukemogenesis in adult human leukemia. Blood.

[b64] Konieczna I, Horvath E, Wang H (2008). Constitutive activation of SHP2 in mice cooperates with ICSBP deficiency to accelerate progression to acute myeloid leukemia. J Clin Investig.

[b65] Yang W, Klaman LD, Chen B (2006). An Shp2/SFK/Ras/Erk signaling pathway controls trophoblast stem cell survival. Dev Cell.

[b66] Ralston A, Rossant J (2006). How signaling promotes stem cell survival: trophoblast stem cells and Shp2. Dev Cell.

[b67] Ren Y, Chen Z, Chen L (2007). Shp2E76K mutant confers cytokine-independent survival of TF-1 myeloid cells by up-regulating Bcl-XL. J Biol Chem.

[b68] Yang Z, Li Y, Yin F (2008). Activating PTPN11 mutants promote hematopoietic progenitor cell-cycle progression and survival. Exp Hematol.

[b69] Cheng HL, Su SJ, Huang LW (2010). Arecoline induces HA22T/VGH hepatoma cells to undergo anoikis - involvement of STAT3 and RhoA activation. Mol Cancer.

[b70] Blechacz BR, Smoot RL, Bronk SF (2009). Sorafenib inhibits signal transducer and activator of transcription-3 signaling in cholangiocarcinoma cells by activating the phosphatase shatterproof 2. Hepatology.

[b71] Morales LD, Casillas Pavon EA, Shin JW (2014). Protein tyrosine phosphatases PTP-1B, SHP-2, and PTEN facilitate Rb/E2F-associated apoptotic signaling. PLoS ONE.

[b72] Yu L, Min W, He Y (2009). JAK2 and SHP2 reciprocally regulate tyrosine phosphorylation and stability of proapoptotic protein ASK1. The Journal of biological chemistry.

[b73] Chan G, Cheung LS, Yang W (2011). Essential role for Ptpn11 in survival of hematopoietic stem and progenitor cells. Blood.

[b74] Nabinger SC, Chan RJ (2012). Shp2 function in hematopoietic stem cell biology and leukemogenesis. Curr Opin Hematol.

[b75] Zhang X, Zhang Y, Tao B (2012). Loss of Shp2 in alveoli epithelia induces deregulated surfactant homeostasis, resulting in spontaneous pulmonary fibrosis. FASEB J.

[b76] Rafiq K, Kolpakov MA, Abdelfettah M (2006). Role of protein-tyrosine phosphatase SHP2 in focal adhesion kinase down-regulation during neutrophil cathepsin G-induced cardiomyocytes anoikis. J Biol Chem.

[b77] Amin AR, Thakur VS, Paul RK (2007). SHP-2 tyrosine phosphatase inhibits p73-dependent apoptosis and expression of a subset of p53 target genes induced by EGCG. Proc Natl Acad Sci USA.

[b78] Lennartsson J, Burovic F, Witek B (2010). Erk 5 is necessary for sustained PDGF-induced Akt phosphorylation and inhibition of apoptosis. Cell Signal.

[b79] Courboulin A, Paulin R, Giguere NJ (2011). Role for miR-204 in human pulmonary arterial hypertension. J Exp Med.

[b80] Li J, Reed SA, Johnson SE (2009). Hepatocyte growth factor (HGF) signals through SHP2 to regulate primary mouse myoblast proliferation. Exp Cell Res.

[b81] Zhu Y, Park J, Hu X (2010). Control of oligodendrocyte generation and proliferation by Shp2 protein tyrosine phosphatase. Glia.

[b82] Li L, Modi H, McDonald T (2011). A critical role for SHP2 in STAT5 activation and growth factor-mediated proliferation, survival, and differentiation of human CD34^+^ cells. Blood.

[b83] Furcht CM, Buonato JM, Skuli N (2014). Multivariate signaling regulation by SHP2 differentially controls proliferation and therapeutic response in glioma cells. J Cell Sci.

[b84] Hu Z, Fang H, Wang X (2014). Overexpression of SHP2 tyrosine phosphatase promotes the tumorigenesis of breast carcinoma. Oncol Rep.

[b85] Rose DW, Xiao S, Pillay TS (1998). Prolonged *vs* transient roles for early cell cycle signaling components. Oncogene.

[b86] Sturla LM, Zinn PO, Ng K (2011). Src homology domain-containing phosphatase 2 suppresses cellular senescence in glioblastoma. Br J Cancer.

[b87] Buet D, Gallais I, Lauret E (2012). Cotargeting signaling pathways driving survival and cell cycle circumvents resistance to Kit inhibitors in leukemia. Blood.

[b88] Peng G, Cao RB, Li YH (2014). Alterations of cell cycle control proteins SHP1/2, p16, CDK4 and cyclin D1 in radioresistant nasopharyngeal carcinoma cells. Mol Med Rep.

[b89] Tsang YH, Han X, Man WY (2012). Novel functions of the phosphatase SHP2 in the DNA replication and damage checkpoints. PLoS ONE.

[b90] Kathpalia VP, Mussak EN, Chow SS (2006). Genome-wide transcriptional profiling in human squamous cell carcinoma of the skin identifies unique tumor-associated signatures. J Dermatol.

[b91] Zhang L, Yang Z, Ma A (2014). Growth arrest and DNA damage 45G down-regulation contributes to Janus kinase/signal transducer and activator of transcription 3 activation and cellular senescence evasion in hepatocellular carcinoma. Hepatology.

[b92] Xu J, Zeng LF, Shen W (2013). Targeting SHP2 for EGFR inhibitor resistant non-small cell lung carcinoma. Biochem Biophys Res Commun.

[b93] Liu W, Yu B, Xu G (2013). Identification of cryptotanshinone as an inhibitor of oncogenic protein tyrosine phosphatase SHP2 (PTPN11). J Med Chem.

[b94] Duan YQ, Ma Y, Wang XJ (2014). Design potential selective inhibitors for treating cancer by targeting the Src homology 2 (SH2) domain-containing phosphatase 2 (Shp2) with core hopping approach. Protein Pept Lett.

[b95] Sharma N, Everingham S, Zeng LF (2014). Oncogenic KIT-induced aggressive systemic mastocytosis requires SHP2/PTPN11 phosphatase for disease progression in mice. Oncotarget.

[b96] Yu B, Liu W, Yu WM (2013). Targeting protein tyrosine phosphatase SHP2 for the treatment of PTPN11-associated malignancies. Mol Cancer Ther.

